# The utility of SPECT-CT in identifying vertebral endplate changes for basivertebral nerve ablation: A retrospective study with MRI comparison

**DOI:** 10.1016/j.inpm.2026.100751

**Published:** 2026-04-04

**Authors:** Eva Kubrova, Justin Schappell, Jeet Patel, Chris Radlicz, Rosalynn R.Z. Conic, Matthew A. Cascio, Robert Pagan-Rosado, Thomas M. Herrera, Jason S. Eldrige, Sebastian Encalada, Sahil Bade, Sohail Bade, Michael Osborne

**Affiliations:** a Pain Medicine Department, Mayo Clinic, Jacksonville, FL, USA; b Pain Medicine Department, Mayo Clinic Health System, Eau Claire, WI, USA; c Department of Radiology, Mayo Clinic, FL, USA; d Division of Physical Medicine and Rehabilitation, Department of Orthopedic Surgery, Washington University in St. Louis, St. Louis, Missouri, USA; e Department of Physical Medicine and Rehabilitation, Mayo Clinic, FL, USA

## Abstract

**Background:**

Vertebrogenic low back pain is an increasingly recognized source of chronic low back pain. Although Magnetic resonance imaging (MRI) remains the primary diagnostic tool for evaluating Modic changes (MC), its ability to confirm symptomatic pain is limited. Nuclear Medicine SPECT/CT may provide additional value by identifying metabolically active endplate pathology.

**Objective:**

The goal of this retrospective study is to characterize suspected correlation between MC identified on MRI with endplate radiotracer uptake on SPECT/CT and to investigate the potential utility of SPECT-CT in evaluating and treating patients with suspected vertebrogenic low back pain.

**Methods:**

A retrospective chart review of patients who underwent BVNA was conducted between March 2020 and July 2024. Patients with both pre-procedural MRI and SPECT/CT were identified, and a correlative descriptive analysis was performed to assess overlap of both imaging methods.

**Results:**

The MRI and SPECT/CT scans of 52 patients were evaluated, with Type 1 Modic changes (MC1) and Type 2 Modic changes (MC2) varying in correlation to SPECT/CT by spinal level. Most notable overlap was found when correlating positive MC1 changes; there were 80.9% patients in whom for each positive MC1, there was a positive SPECT/CT correlate. The PPV of SPECT/CT was 62.1% and the NPV was 95.2% for MC1. The PPV of SPECT/CT was 54.3% and the NPV was 89.1% for MC2. Complete (100%) MC1, MC2 and SPECT/CT level overlap was seen in 44% of patients. The Cohen's kappa measurement of agreement between the MRI and SPECT/CT was 70.1%.

**Conclusion:**

This study demonstrates a high MC1 and moderate MC2 correlation with increased endplate radiotracer uptake on SPECT/CT in patients selected for BVNA. These findings suggest that while SPECT/CT may complement or potentially substitute for MRI in evaluation of patients with suspected vertebrogenic low back pain, its role as a standalone alternative diagnostic tool remains uncertain.

## 1 Introduction

Chronic low back pain (CLBP) is one of the most prevalent and disabling health conditions, affecting over 30 million Americans and placing a substantial burden on the healthcare system and negatively impacting workplace productivity [1]. In the United States alone, the economic impact of CLBP exceeds $100 billion annually [2]. Common sources of chronic axial low back pain include lumbosacral facet joints, sacroiliac joints, and intervertebral discs [3]. Recent studies have identified vertebral endplates via basivertebral nerve innervation as a distinct pain entity, now referred to as *vertebrogenic pain* [4,5].

Vertebrogenic pain originates from structural and inflammatory changes at the vertebral endplates, which are often visualized on magnetic resonance imaging (MRI) as Modic changes (MCs). MCs are classified into three types: Type 1 (edematous/inflammatory), Type 2 (fatty), and Type 3 (sclerotic). Type 1 changes appear hypointense on T1-weighted (T1W) MRI sequences and hyperintense on T2-weighted (T2W) sequences. Type 2 changes are hyperintense on both T1W and T2W, while Type 3 changes appear hypointense on both T1W and T2W [6].

Systematic reviews have shown a positive association between CLBP and types 1 and 2 MC, suggesting that MCs may serve as a biomarker for pain originating from endplate pathology [7,8]. Immunohistochemical analysis of the basivertebral nerve has revealed high concentrations of nociceptive and inflammatory markers, such as substance P, calcitonin gene-related peptide, and protein gene product 9.5, further underscoring its role in vertebrogenic low back pain and vertebral endplate pain transmission [6].

MRI remains the gold standard imaging modality for evaluating CLBP and is valuable for detecting MCs and endplate defects. However, its ability to determine whether these findings are symptomatic is limited, as many degenerative changes are also present in asymptomatic individuals [8]. In contrast, single-photon emission computed tomography combined with computed tomography (SPECT/CT) provides both anatomical and metabolic information, potentially improving the identification of pain generators in degenerative spine disease by detecting increased radiotracer uptake in metabolically active endplates [9].

There have been two studies which evaluated the correlation between MCs and SPECT/CT. First, a prospective study found a 96% correlation between Type 1 MCs and high metabolic activity on SPECT/CT, suggesting that SPECT/CT may help diagnose inflammatory endplates [9]. Conversely, a large retrospective cohort study found significant correlations between SPECT/CT positivity and advanced disc degenerative changes (based on the Pfirrmann classification) and endplate degenerative changes (Rajasekaran classifications); however, no strong association was observed between MCs and SPECT/CT activity [10]. This may suggest that increased endplate activity on SPECT/CT may be more closely related to intervertebral disc degeneration and endplate destruction than to subchondral bone marrow changes. The lack of association may indicate that the mechanisms driving SPECT/CT positivity differ from those underlying MCs.

The goal of this retrospective study is to correlate MCs identified on MRI with radiotracer uptake in vertebral endplates on SPECT/CT in patients undergoing Basivertebral Nerve Ablation (BVNA). Randomized controlled trials have demonstrated the safety, efficacy, and durability of BVNA in treating vertebrogenic CLBP, providing a strong evidence base for its clinical use [11]. Type 1 or Type 2 MCs on MRI are required under current guidelines to qualify for BVNA. Establishing whether SPECT/CT can serve as a functional biomarker for symptomatic endplate pathology could refine patient selection for BVNA, enhance diagnostic accuracy, and ultimately improve clinical outcomes in vertebrogenic CLBP. Furthermore, it may demonstrate SPECT/CT's role as a viable alternative for patients unable to undergo MRI when suspicion of vertebrogenic low back pain is high.

## 2 Methods

After obtaining an Institutional Review Board (IRB) approval (IRB number: 24-011-024) of the study protocol, we performed a retrospective chart review of patients who underwent BVNA at our institution at Mayo Clinic Florida.

### 2.1 Inclusion and exclusion criteria

Adult patients who underwent BVNA performed by pain medicine or interventional radiology physicians between March 2020 and July 2024 were included. Patients had to have imaging modalities of interest (lumbar spine MRI and SPECT/CT) prior to undergoing BVNA to be included in the study.

### 2.2 Basivertebral nerve ablation procedure

BVNA was performed using The Intracept™ Intraosseous Nerve Ablation System (Boston Scientific, Minneapolis, MN, US; previously a device owned by Relievant). BVNA is intended to ablate the basivertebral nerves of vertebral bodies L3 through S1 and is indicated for patients experiencing chronic low back pain lasting six months or more who have not responded to at least six months of conservative care. Patients require MRI evidence of MC1 or MC2 in order to qualify for the procedure. Targeting levels above L3 is considered off label. The decision-making process in evaluation for BVNA involves clinical correlation of imaging findings with history and physical examination.

### 2.3 Primary and secondary outcomes

The primary goal of the study was to evaluate the correlation of SPECT/CT imaging with MRI findings of MC1 and MC2. We evaluated the accuracy of SPECT/CT in predicting positive Modic changes, and vice versa. A secondary aim was to investigate whether SPECT/CT could aid in decision-making for determining levels eligible for BVNA, particularly in cases where MRI is contraindicated or insufficient for assessing endplate changes. In accordance with current clinical recommendations, target levels for BVNA were chosen in most cases based on Modic findings on MRI alone.

### 2.4 Imaging review

A board-certified Neuroradiologist (JP) reviewed all cases to identify Modic type 1, 2 and 3 changes on preprocedure MRI, as well as increased vertebral endplate radiotracer uptake on SPECT/CT imaging.

Modic changes were assessed on sagittal MRI sequences, including 1) a sagittal T1-weighted sequence AND 2) either a sagittal T2-weighted sequence, fat-suppressed T2-weighted sequence or a sagittal Short tau inversion recovery (STIR) sequence. The choice between fat-suppressed T2W and STIR sequences varied depending on the technique used.

Modic changes were deemed present if signal alterations were observed along the endplates on each side of the intervening disc space. For example, L4-L5 MC required the signal change to be along the inferior endplate of L4 and superior endplate of L5.

MC1 were identified by increased signal intensity along the endplates on fat-suppressed T2-weighted images or STIR images relative to the background marrow signal, indicating reactive marrow edema. MC2 were identified if there was increased signal intensity along the endplates on T1-weighted images compared to the background marrow signal of the vertebral body indicating reactive fatty marrow change. MC3 changes were identified in one patient and are defined by hypointense signals on both T1 and T2-weighted images relative to background marrow indicating reactive sclerotic change. In cases of levels with overlapping MC1 and MC2 changes, the level was labeled as both MC1 and MC2 positive.

**Fig. 1:**
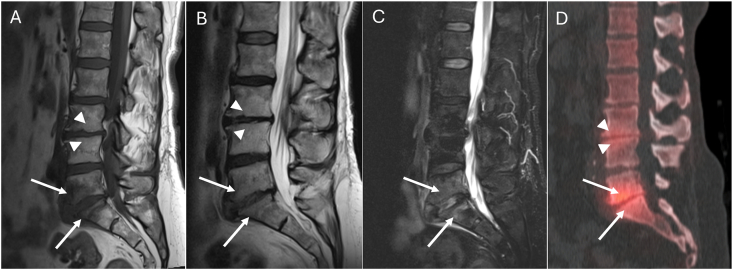
Example of concordance between MRI and SPECT-CT. Sagittal T1-weighted (A), sagittal T2-weighted (B) and STIR sequence (C) images demonstrate Modic type 1 changes at L5-S1 (arrows) and Modic type 2 changes at L3-L4 (arrowheads), with associated reactive changes on sagittal reformatted bone SPECT-CT (D) fused image. At L5-S1, Modic type 1 changes are indicated by hypointense signal in the vertebral bodies adjacent to the L5-S1 disc space on the T1-weighted image, with hyperintense signal on the T2-weighted sequence and STIR image consistent with marrow edema, and there is associated increased radiotracer uptake compared to the background signal in the rest of the vertebrae. At L3-L4, Modic type 2 changes are indicated by hyperintense signal in the vertebral bodies adjacent to the L3-L4 disc space on the T1-weighted image, hyperintense signal on the T2-weighted image consistent with fatty marrow change compared to the background signal in the rest of the vertebrae, and there is associated increased radiotracer uptake as well.

**Fig. 2:**
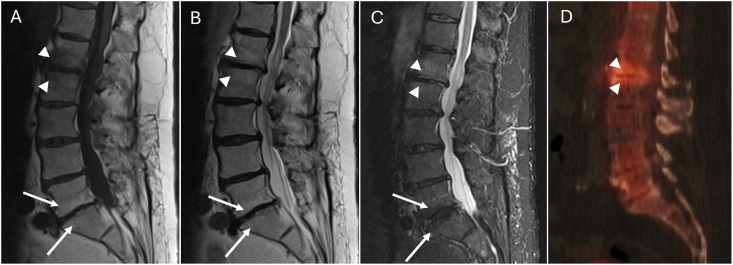
Example of partial discordance between MRI and SPECT-CT. Sagittal T1-weighted (A), sagittal T2-weighted (B) and STIR sequence (C) images demonstrate Modic type 1 changes at L1-L2 (arrowheads) and Modic type 2 changes at L5-S1 (arrows). At L5-S1, Modic type 2 changes are indicated by hyperintense signal in the vertebral bodies adjacent to the L5-S1 disc space on the T1-and T2-weighted images, with hypointense signal on the STIR image consistent with fatty replacement, and there is no significant associated radiotracer uptake. At L1-2, Modic type 1 changes are indicated by hypointense signal in the vertebral bodies adjacent to the L1-L2 disc space on the T1-weighted image, and hyperintense signal on the T2-weighted image consistent with marrow edema compared to the background signal in the rest of the vertebrae. At L1-L2 there is associated increased radiotracer uptake on sagittal reformatted bone SPECT-CT fused image (D).

Reactive changes on SPECT/CT were assessed by review of sagittal fused SPECT and CT images. SPECT/CT changes were considered present or positive if increased radiotracer uptake was seen along the endplates relative to the background signal of the marrow in the remainder of the vertebral body. These findings were then compared with MRI findings to assess correlation and degree of overlap (Fig. 1, Fig. 2).

### 2.5 Statistical analysis

We performed descriptive statistical analysis, which included the percentage of patients with overlapping levels positive for MCs or increased SPECT/CT uptake, stratified by spine levels. Positive predictive value (PPV) and negative predictive value (NPV) were calculated to assess the diagnostic performance of SPECT/CT compared to MRI as the gold standard. PPV and NPV analysis of MRI compared to SPECT/CT as the gold standard was also performed. Cohen's kappa was calculated to assess the agreement between the two tests.

## 3 Results

A total of 52 patients who completed both lumbar spine MRI without intravenous contrast and SPECT/CT imaging before BVNA were identified through a retrospective chart review. Median patient age was 76 (IQR: 68–82) and 38.5% were females. Median time between MRI and SPECT/CT was 3 months (IQR = 9, range 0-60 months) and average 1month.

There were a total of 364 levels reviewed, with n = 84 levels with MC1 changes, n = 90 levels with MC2 changes. In some cases, MC1 and MC2 overlap was present. Increased SPECT-CT uptake was found in 116 levels (Table 1).

**Table 1:** – MRI with Modic 1 or Modic 2 changes and SPECT/CT changes; n - number of spinal levels.

Level	MRI - Modic 1 (n)	MRI - Modic 2 (n)	SPECT-CT (n)
**T12-L1**	4	3	2
**L1-L2**	12	7	15
**L2-L3**	18	20	21
**L3-L4**	13	18	25
**L4-L5**	16	18	23
**L5-S1**	20	23	28
**S1-S2**	1	1	2
**Total**	84	90	116

Based on these imaging findings and pertinent clinical findings, BVNA was performed most commonly targeting 2 vertebral body levels (n = 21), followed by 3 levels (n = 12), 4 levels (n = 8), 5 levels (n = 7) and 6 levels (n = 4).

### 3.1 Association between modic changes and positive SPECT/CT

The distribution of positive MC1, MC2 and SPECT/CT varied across individuals, with some demonstrating more levels with positive MC, while others demonstrated more positive levels on SPECT/CT. Notably, some levels also had overlapping MC1 and MC2 (mixed changes).

There were a total of 23/52 subjects (44%) in whom there was a 100% overlap of SPECT/CT and Modic changes (without any outstanding positive MC 1, MC 2 or SPECT/CT changes). When calculated per individual vertebral body level, there was 81.8% overlap of SPECT/CT with either MC1 or MC2 (ranged 0-100%).

For MC1 comparison with SPECT/CT, there were 38/47 subjects (80.9%, 72 levels) in whom for each positive MC1, there was a positive SPECT/CT change. There were 26/52 (50%) subjects where for each positive SPECT/CT level, there was a positive MC1. The SPECT/CT missed 12/84 (14.3%) total levels in patients that had positive MC1 on MRI and identified additional 44 levels. The positive predictive value (PPV) of SPECT/CT using MRI as the gold standard was 62.1% and the negative predictive value (NPV) was 95.2% (Table 2.). For MRI, using SPECT/CT as the theoretical gold standard the PPV was 85.7% and NPV was 84.3%.

**Table 2:** Positive predictive value (PPV) and negative predictive value (NPV) of SPECT/CT compared to gold standard imaging (MRI) for Modic 1, Modic 2 and combined changes.

Predictive value of SPECT/CT	Modic 1	Modic 2	Modic 1 or Modic 2
**PPV**	62.1%	54.3%	87.1%
**NPV**	95.2%	89.1%	86.3%

For MC2 comparison with SPECT/CT, there were only 18/37 subjects (48.6%, 63 levels) where for each positive MC2 there was a positive SPECT/CT change. There were 18/52 subjects (34.6%) where each positive SPECT/CT level corresponded to positive MC2.

The SPECT/CT missed 27/90 (30%) total levels in patients that had positive MC2 on MRI, and identified 53 additional levels. The PPV of SPECT/CT using MRI as the gold standard was 54.3% and the NPV was 89.1% (Table 2.). For MRI, using SPECT/CT as the theoretical gold standard the PPV was 70% and NPV 80.7%.

Therefore, there was a higher chance of positive SPECT/CT to show also positive MC1 rather than MC2 (50% subjects vs 34.6% subjects).

Overall, there were 29/51 (56.9%) subjects and 101 total levels where each positive SPECT/CT level corresponded with MRI evidence of MC1 and/or MC2. The SPECT/CT missed 34/132 levels and identified additional 15 levels. The PPV of SPECT/CT was 87.1% and the NPV was 86.3% (Table 2.). For MRI, the PPV was 74.8% and NPV was 93.4%. The Cohen's kappa measurement of agreement between the MRI and SPECT/CT was 70.1%.

## 4 Discussion

Our analysis informs the evolving role of SPECT/CT as a functional imaging adjunct to MRI in the evaluation of vertebrogenic low back pain. MRI is currently a gold standard imaging modality to determine levels targeted for BVNA. Our hypothesis included utilization of SPECT/CT as a predictor of positive MC to help inform levels targeted for BVNA in cases where MRI is not feasible or inconclusive. We found that while the overlap between MCs and SPECT/CT uptake was moderate, the concordance was notably stronger for MC1 compared to MC2. Specifically, SPECT/CT was less likely to miss MC1 (14.3%) compared to MC2 (34.6%) across the levels evaluated. The ability of SPECT/CT to predict positive MC1 was moderate, while its ability to predict MC2 was lower (PPV 62.1% vs 54.3%), however, the overall PPV for both MC was high at 87.1%.

These findings support the proposed inflammatory pathophysiology of MC1, which reflects active marrow edema and inflammation associated with increased bone turnover. In contrast, MC2 are believed to represent fatty marrow replacement and decreased bone formation, typically indicating more stable, chronic changes [12]. Furthermore, elevated serum C-reactive protein levels have been reported in patients with chronic low back pain with mainly MC1, whereas lower C-reactive protein levels are seen in conjunction with MC2, corroborating this distinctive inflammatory state [13]. Similarly, levels of proinflammatory cytokines, such as tumor necrosis factor, are higher in endplates with MC1 compared to MC2 [14]. Histopathologic studies have confirmed that these endplates are richly innervated with nociceptive fibers containing substance P and CGRP, which reinforces the mechanistic rationale for the detection of such metabolically active changes via functional imaging modalities like SPECT/CT [15,16].

While lower than the concordance observed in patients with MC1, SPECT/CT still demonstrated significant overlap in subjects with MC2. MC2 are generally considered more stable, but there is growing evidence that some MCs may harbor low-grade inflammation. This is supported by MRI studies in which many MC2 endplates failed to show signal suppression on fat-saturated sequences, suggesting incomplete fatty replacement [17]. Moreover, MC2 have been associated with systemic metabolic dysregulation, due to their association in patients with higher fat mass and lower fat-free mass [18].

Some vertebral levels in our study also exhibited overlapping MC1 and MC2. Apart from the overlap of distinct MC in a mixed picture, it is important to note that although the typical natural history involves progression from MC1 to MC2, reverse conversion is also possible. In such cases, renewed degeneration and inflammation can result in the reappearance of superimposed MC1 on previously stable MC2 [[19], [20], [21]]. Importantly, MC1 are generally more dynamic than MC2, with new or progressive changes more strongly associated with pain. In contrast, transitions to MC2 often occur without concurrent worsening of symptoms [22,23]. These observations further highlight the value of functional imaging like SPECT/CT in identifying clinically relevant, metabolically active changes that may otherwise be missed or underestimated on structural imaging alone.

Another important implication of our findings is their relevance for refining patient selection in real-world practice. Prior studies have not directly compared outcomes of BVNA between patients with MC1 and MC2 [[24], [25], [26], [27]]. Given our findings of higher SPECT/CT correlation with MC1 and the distinct pathophysiologic profiles of the two types, future studies should evaluate the differential efficacy of BVNA by Modic subtype.

One study used both the presence of MC on MRI and focal vertebral body uptake on SPECT/CT as criteria to proceed to BVNA, along with negative medial branch blocks to exclude zygapophyseal joint pain [28]. In this cohort, 96.5% experienced clinically meaningful reductions in pain and disability. These outcomes appear improved compared to prior MRI-only studies, with a mean VAS reduction at 12 months similar to that reported by Macadaeg et al. (∼4.3 cm) and greater than that in the Fischgrund et al. trial (∼3.7 cm) [1,24]. Furthermore, the proportion of patients achieving the MCID for VAS was lower in the MRI-only studies. However, the study's use of SPECT/CT is not clearly described and may have had limited impact on decision making about BVNA levels. Incorporation of a diagnostic medial branch block is another potential confounding factor, thereby potentially contributing to the positive observed clinical outcome. Nevertheless, adding SPECT/CT to the diagnostic algorithm could help clarify whether ambiguous endplate signal changes truly represent metabolically active pain sources which can optimize procedural targeting and possibly improve outcomes. Similar strategies have been applied to fusion surgeries, where SPECT-positive cervical or lumbar segments have guided improved surgical selection [19,20].

More than three quarters of the spinal levels in our cohort that were SPECT/CT positive had a corresponding change of MC1, MC2, or both. An earlier study by Lusins et al. demonstrated SPECT positivity in 37 of 38 patients with MC1 or MC2, with authors proposing its utility of SPECT in identifying early marrow changes before MCs are appreciated on MRI [29]. Compared to planar bone scintigraphy, SPECT has higher sensitivity for spinal pathology because it enables multiplanar localization [30].The multimodality of SPECT with CT enhances both morphological and physiological assessment, allowing precise localization of metabolically active regions [30]. However, specificity remains a limitation, as tracer uptake depends on osteoblastic activity and general bone remodeling rather than inflammation alone.

Prior work has shown high overlap between bone scintigraphy tracer uptake and MC1 changes, with 93% showing uptake when MC1 was present and 64% when other types coexisted with MC1. In contrast, only 5% of other types and combinations (mixed type 2 and 3) changes showed uptake, reinforcing the strong link between bone turnover and MC1 [31]. In the Russo et al. study, SPECT/CT identified metabolic activity in 96.1% of MC1, 77.8% of MC2, and 56% of MC3 endplates [9]. While our study similarly demonstrated higher SPECT/CT positivity in MC1 compared to MC2, the percentage of SPECT/CT positivity for all three MC subtypes was lower than that found in Russo's study.

Clinically, our results suggest that SPECT/CT may be uniquely valuable in confirming metabolically active MCs, especially when MRI findings are ambiguous or when multiple pain generators are present. Structural imaging alone is insufficient to fully characterize vertebral endplate changes, especially since MCs can occur asymptomatically [19]. By detecting bone remodeling activity, SPECT/CT may help differentiate metabolically quiescent from actively inflamed endplates that are more likely to be pain generators.

Overall, SPECT/CT failed to detect a proportion of Modic-positive levels identified on MRI, suggesting that while it is capable of identifying metabolically active vertebral endplate changes, its sensitivity as a standalone diagnostic tool may be limited. Nevertheless, in patients unable to undergo MRI, SPECT/CT may represent a viable alternative imaging modality for evaluating BVNA candidacy, given its relatively high NPVs, especially for MC1. Therefore, a negative SPECT/CT level has a high likelihood of being MRI negative with respect to MC1 and MC2.

Regarding the assessment of our clinical outcomes, which was not a primary goal of the study, we note that the vertebral level targeting in our cohort was based primarily on MRI findings, in accordance with current accepted clinical practice. SPECT/CT was used to support and guide clinical decision-making only as needed or to rule out other clinically relevant pain generators such as facet-joint related pain, interspinous process pain, sacroiliac joint pain and other. As such, we were unable to assess the full predictive utility of SPECT/CT in directing treatment or evaluating response. However, we note that the value of SPECT/CT as a supplementary diagnostic tool to MRI may be valuable in this context.

## 5 Limitations

This study has several limitations that should be considered when interpreting its findings. First, the study was conducted at a single tertiary academic medical center, which may limit the generalizability of the results to other clinical settings. Additionally, all imaging studies were interpreted by a single board-certified neuroradiologist, introducing the potential for observer bias. The retrospective nature of the image review may also contribute to discrepancies between the interpretations used in this analysis and those that originally informed clinical decision making. The variability in the timing of imaging—specifically, a median lag of three months between SPECT/CT and MRI—may have introduced temporal bias, potentially affecting the observed concordance between modalities.

The relatively smaller sample size may limit the detection of more nuanced relationships between MRI and SPECT/CT findings. This cohort also consisted solely of patients who underwent BVNA, which may impact the PPV and NPV and introduce a selection bias. Furthermore, we did not include patient-reported outcomes or post-procedural follow-up data, preventing us from correlating imaging characteristics with clinical response. Thus, while we were able to quantify the imaging overlap between modalities, the clinical relevance of these findings remains uncertain and an opportunity for further investigation.

Future studies should aim to validate these findings in larger, multicenter cohorts with standardized imaging acquisition and interpretation protocols. Trials should combine MRI, SPECT/CT, and standardized outcomes to validate our findings and define the optimal diagnostic pathway for vertebrogenic low back pain.

## 6 Conclusions

Our retrospective analysis revealed that SPECT/CT demonstrated partial but inconsistent overlap with Modic changes (MC1 and MC2) identified on MRI in patients undergoing basivertebral nerve ablation (BVNA) for chronic low back pain. Although SPECT/CT showed strong overall concordance with any positive MC1 or MC2 findings (PPV 87.1%), and a low miss rate for MC1 (14.3%, NPV 95.2%), it still missed MC2 lesions in 34.6% of levels. These results suggest that SPECT/CT may offer a complimentary and perhaps best alternate diagnostic modality to MRI in identifying vertebral levels for BVNA in patients with suspected vertebrogenic low back pain.
